# The *Chlamydia trachomatis* type III-secreted effector protein CteG induces centrosome amplification through interactions with centrin-2

**DOI:** 10.1073/pnas.2303487120

**Published:** 2023-05-08

**Authors:** Brianna Steiert, Carolina M. Icardi, Robert Faris, Paige N. McCaslin, Parker Smith, Aloysius J. Klingelhutz, Peter M. Yau, Mary M. Weber

**Affiliations:** ^a^Department of Microbiology and Immunology, University of Iowa Carver College of Medicine, Iowa City, IA 52242; ^b^Carver Biotechnology Center–Protein Sciences Facility, University of Illinois at Urbana–Champaign, Urbana, IL 61801

**Keywords:** chlamydia, effector, type III secretion, centrosome

## Abstract

The centrosome is the main microtubule organizing center of the cell and is crucial for mitotic spindle assembly, chromosome segregation, and cell division. Centrosome duplication is tightly controlled, yet several pathogens, most notably oncogenic viruses, perturb this process leading to increased centrosome numbers. Infection by the obligate intracellular bacterium *Chlamydia trachomatis* (*C.t.*) correlates with blocked cytokinesis, supernumerary centrosomes, and multipolar spindles; however, the mechanisms behind how *C.t.* induces these cellular abnormalities remain largely unknown. Here we show that the secreted effector protein, CteG, binds to centrin-2 (CETN2), a key structural component of centrosomes and regulator of centriole duplication. Our data indicate that both CteG and CETN2 are necessary for infection-induced centrosome amplification, in a manner that requires the C-terminus of CteG. Strikingly, CteG is important for in vivo infection and growth in primary cervical cells but is dispensable for growth in immortalized cells, highlighting the importance of this effector protein to chlamydial infection. These findings begin to provide mechanistic insight into how *C.t.* induces cellular abnormalities during infection, but also indicate that obligate intracellular bacteria may contribute to cellular transformation events. Centrosome amplification mediated by CteG–CETN2 interactions may explain why chlamydial infection leads to an increased risk of cervical or ovarian cancer.

## Significance

The presence of more than two centrosomes is a hallmark of many types of cancer, including cervical and ovarian cancers of which *Chlamydia trachomatis* (*C.t.*) infection is a significant risk factor. Despite the importance of this problem, how *C.t.* orchestrates these drastic changes in the host cell remains poorly understood. Here, we describe how *C.t.* uses the effector protein CteG to drive centrosome amplification via manipulation of a key regulator of centriole duplication, centrin-2. This work begins to define how *C.t.* induces centrosome amplification to promote its replication while potentially contributing to devastating long-term negative consequences for normal host physiology. Furthermore, it may help elucidate why chlamydial infection leads to an increased cancer risk.

The centrosome is the main microtubule organizing center (MTOC) of the cell and is involved in mitotic spindle assembly, chromosome segregation, cell division, microtubule structure, and cell shape (1). The centrosome is comprised of two barrel-shaped centrioles that are embedded in a matrix of proteins known as the pericentriolar material. Centrosomes duplicate only once per cycle, initiating the process at the G1/S transition, and completing this process prior to entry into mitosis (2). Given the intimate link between cell cycle progression and centrosome duplication, there is increasing support for the notion that the centrosome itself is a key regulator of the cell cycle (3).

Centrosome abnormalities are hallmarks of numerous types of human cancers and correlate with tumorigenesis and poor patient outcomes (4). Centrosome amplification, caused by cell–cell fusion, dysregulation of centrosome duplication, or cytokinesis defects (5), leads to increased genomic instability, resulting in aneuploidy and chromosomal instability (6). Centrosome amplification has also been shown to be sufficient to cause tumorigenesis in flies and mammals (7, 8). Typically, increased centrosome number alters mitotic spindle formation, leading to multipolar spindles, which can support tumorigenesis by promoting merotelic attachments and chromosome mis-segregation (9, 10). Division in cells with multipolar spindles can be deleterious, but cancer cells overcome this fitness cost by clustering extra centrosomes to achieve bipolar mitosis (5, 11). Oncogenic viruses, such as human papillomavirus (HPV) and Epstein–Barr virus (EBV), similarly induce centrosome abnormalities (12). Cervical cancers, associated with high-risk HPV infection, are characterized by multipolar spindles, which is linked to abnormal centrosome number (13). The HPV oncoprotein E7 induces centrosome amplification by targeting centriole duplication, which can lead to centrosome accumulation and ultimately causes genomic instability. Similarly, EBV infection leads to overproduction of centrosomes through its BNRF1 protein (14).

*Chlamydia trachomatis* (*C.t.*) is an obligate intracellular bacterium that is the etiological agent of multiple human diseases (15). Importantly, current or prior chlamydia infection is associated with an increased risk of development of ovarian and cervical cancers (16, 17). Chlamydia is known to cause host cell transformation and it has been speculated that *C.t.*-induced changes to the host cell linger after clearance of infection (18), potentially explaining why chlamydial infection increases the risk of developing certain types of cancers. Early during infection, *C.t.* traffics along microtubules to the MTOC of the cell to establish its intracellular niche, termed the inclusion. Here it initiates and maintains a close association with the MTOC/centrosomes (19). Studies have shown that chlamydia infection leads to supernumerary centrosomes, mitotic spindle defects, multinucleation, aneuploidy, and blocked cytokinesis (18, 20–24). In *C.t.* infection models, centrosome amplification has been attributed to both cytokinesis defects and dysregulation of the centrosome duplication machinery (20, 21, 25). While the initial observations that *C.t.* infection induces host cellular abnormalities were made over 15 y ago, how *C.t.* orchestrates these cellular changes from the confines of its inclusion remains largely unknown.

As an obligate intracellular pathogen, *C.t.* must establish a niche within a host to proliferate and cause disease. Essential to this intracellular lifestyle is the secretion of over 100 effector proteins, which are delivered through its type III secretion system (T3SS) (26, 27). These effector proteins have been shown to play roles in invasion, nutrient acquisition, and immune evasion (26, 27), but the function of most remains unknown. The **C*hlamydia *t*rachomatis* effector associated with the Golgi (CteG) is a T3SS effector that was previously shown to localize to the Golgi (16 to 30 h postinfection) or plasma membrane (30 to 40 h postinfection) depending on the stage of the infection cycle, but mRNA for CteG is detected as early as 2 h postinfection (28). When expressed in yeast, CteG causes a vacuolar protein sorting defect (28); however, the molecular function of CteG remains unknown.

In this study, we sought to elucidate the molecular function of CteG and its role during *C.t.* infection. We detected an interaction between CteG and the host protein, centrin-2 (CETN2), and further demonstrate that binding requires the C-termini of both proteins. Significantly, our results indicate that CteG is necessary for centrosome amplification but is dispensable for multinucleation and centrosome positioning at the inclusion. Intriguingly, while CteG is dispensable for growth in immortalized cell lines (HeLa and A2EN), the absence of CteG impairs chlamydia’s ability to replicate efficiently in primary cervical cells and in a murine model of *C.t.* infection. Taken together, our data indicate that CteG is important for chlamydial growth and serves as a primary contributor to *C.t-*induced centrosome amplification via manipulation of CETN2.

## Results

### CteG Toxicity in Yeast Is Suppressed by Overexpression of the Anaphase Promoting Complex Subunit 2 (APC2).

**Fig. 1.**
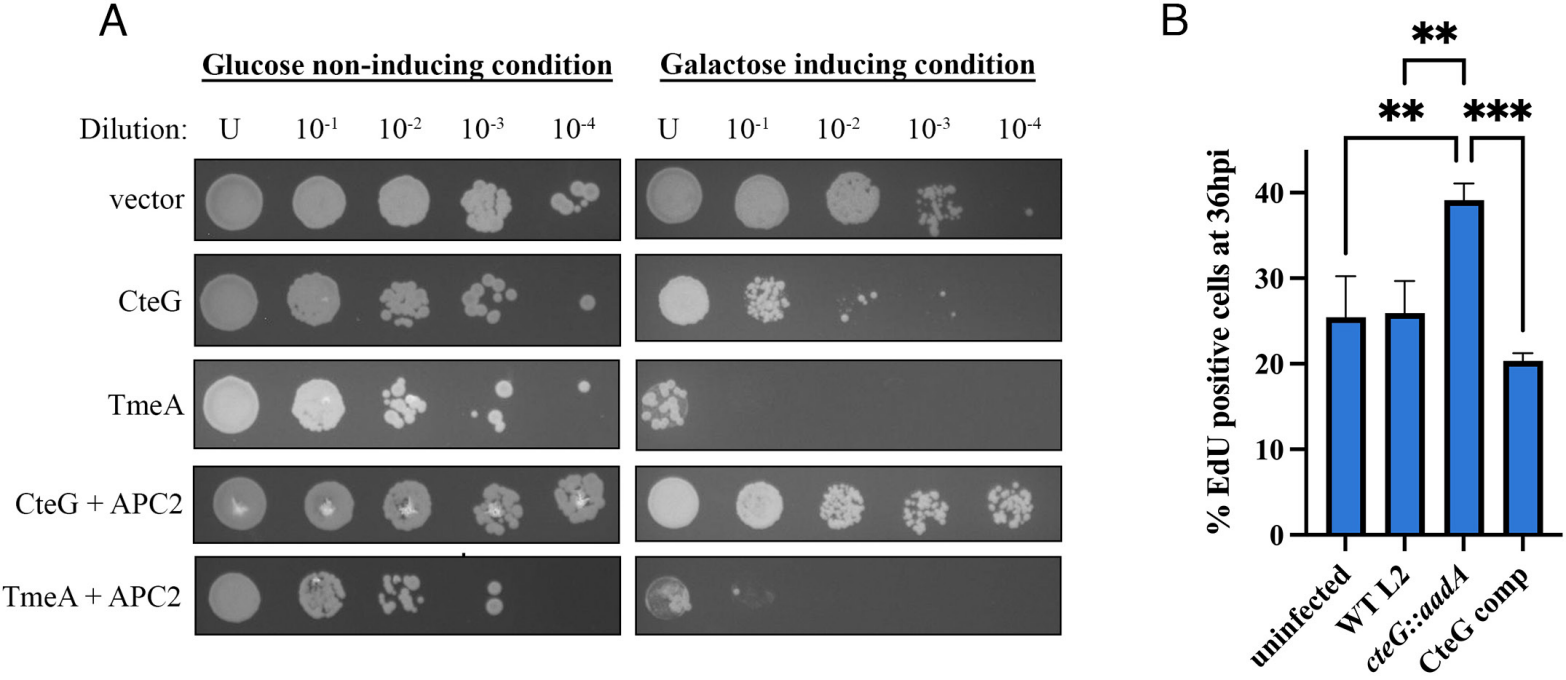
CteG toxicity in yeast is uniquely suppressed by APC2 and contributes to altered cell cycle progression. (*A*) *C.t.* effectors and APC2 were placed under the control of galactose inducible promoters. Transformed yeast were serially diluted and spotted onto glucose- or galactose-containing media to assess toxicity. (*B*) Quantification of EdU-positive HeLa cells 36 h postinfection. Significance was determined using one-way ANOVA followed by Tukey’s multiple comparisons test. Error bars are SD, **P < 0.01, ***P < 0.001. Data are representative of two replicates.

Previous studies demonstrated that expression of CteG in yeast resulted in a vacuolar protein sorting defect (28); however, the precise mechanism and function remain unknown. Here, we exploited yeast genetics to identify the host pathway(s) targeted by CteG. In line with previous observations (29), we demonstrate that when CteG is overexpressed in yeast, a clear toxic phenotype is observed (Fig. 1*A*), suggesting that CteG perturbs an essential host pathway. To identify the target pathway(s), we employed a yeast suppressor screen (30–33). Introduction of a yeast genomic library into the CteG-expressing yeast strain yielded 69 putative suppressor colonies, of which 25 markedly reduced CteG-induced toxicity. When expressed independently, APC2, a subunit of the anaphase promoting complex (APC) that is involved in degradation of cyclins to promote the progression of the cell cycle (11), was found to be sufficient to suppress CteG toxicity in yeast. Importantly APC2 was unable to suppress the toxicity of TmeA (Fig. 1*A*), a T3SS effector previously shown to target N-WASP (34, 35). The yeast suppressor screen provides putative targeted pathway(s), not direct interacting partners, so these data suggest that CteG targets pathway(s) involved in the host cell cycle.

### Cells Infected with a CteG Null Mutant Show Altered Cell Cycle Progression.

To determine whether CteG plays a role in perturbing the host cell cycle, we labeled cells with EdU, which incorporates into actively replicating DNA to mark cells in S phase. Cells were either left uninfected or infected with wild-type (WT) L2, CteG null mutant (*cteG::aadA*) (28), or *cteG::aadA* complemented with pBomb4-tet-CteG-FLAG (CteG comp). At 36 h postinfection, a higher percentage of *cteG::aadA-*infected cells were in S-phase, indicating that these cells are progressing faster through the host cell cycle compared to WT or CteG comp-infected cells (Fig. 1*B*). This suggests that CteG’s role within the host may influence progression of the host cell cycle, either directly, or downstream of the effects of its direct target.

### CteG Binds to the Host Protein, CETN2 during *C.t.* Infection.

**Fig. 2.**
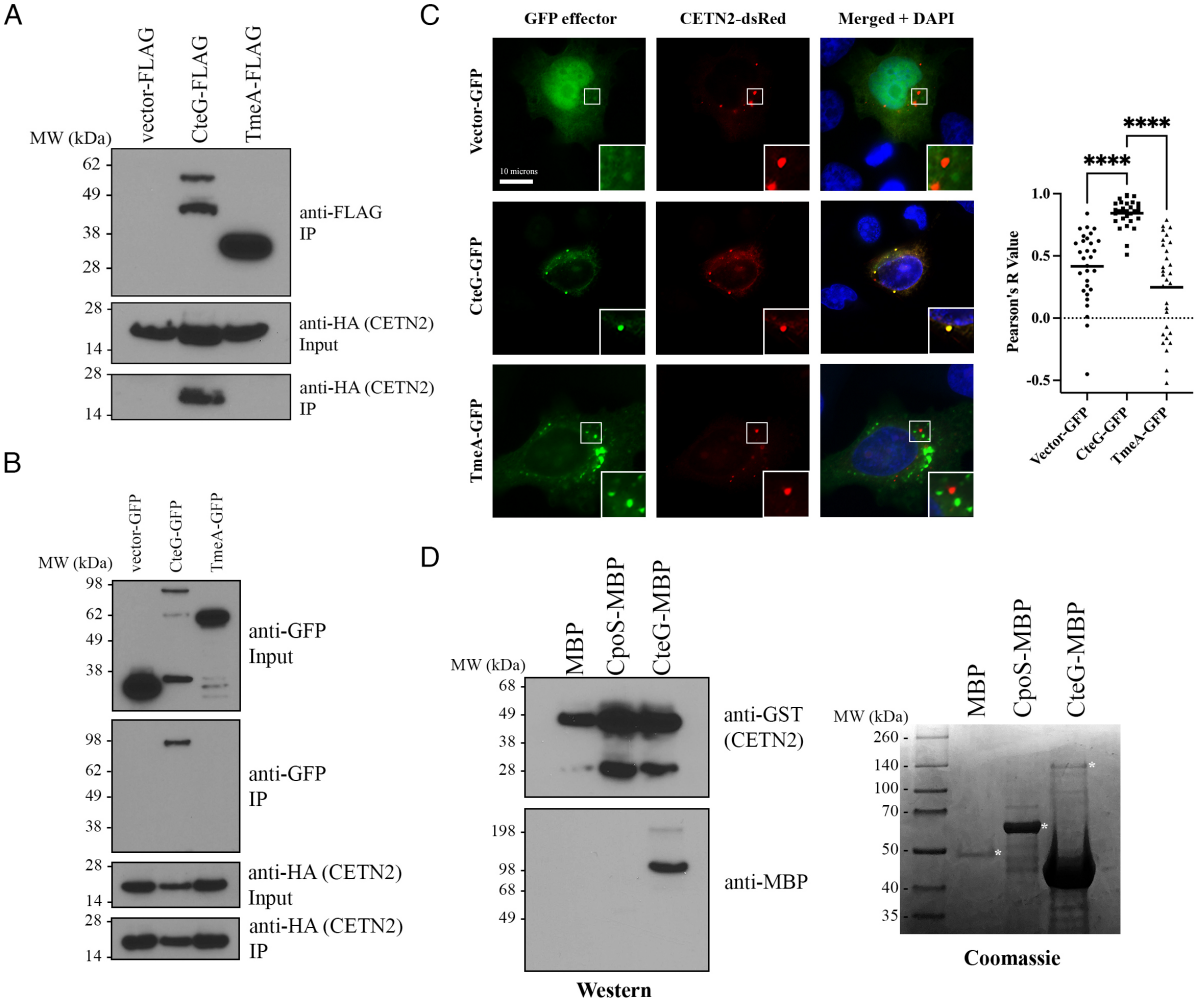
CteG interacts with CETN2 under infection and transfection conditions. (*A*) Co-IP of FLAG-tagged effectors from CETN2-HA transfected HeLa cells infected with *C.t.* expressing the FLAG-tagged effectors. (*B*) Co-IP of HA-tagged CETN2 from HeLa cells cotransfected with GFP-tagged effectors and CETN2-HA. (*C*) Immunofluorescence images of HeLa cells cotransfected with CETN2-dsRed (red) and empty vector-, CteG-, or TmeA- GFP (green). Nucleus is stained with DAPI (blue) (Scale bar, 10 microns). Pearson Correlation Coefficients (R Value) were calculated using ImageJ Coloc2 function. Significance was determined using one-way ANOVA followed by Tukey’s multiple comparisons test. Error bars are SD, ****P < 0.0001. (*D*) Co-IP of purified CETN2-GST and MBP-tagged effectors proteins. Coomassie showing expression of MBP tagged proteins. * indicate correct molecular weight. Data are representative of two to three replicates.

To identify physiologically relevant targets of CteG, we employed affinity purification-mass spectrometry (AP-MS). Expression was confirmed prior to AP-MS analysis by western blotting (*SI Appendix*, Fig. S1). For analysis of MS data, FLAG-tagged CteG results were compared to FLAG-tagged empty vector and only hits with greater than 1 peptide count that were unique to CteG were considered for further analysis, leaving 76 putative targets (*SI Appendix*, Table S2). With an average unique peptide count of 9 and an average of 21 matches across three biological replicates, a fragment of CETN2 was the second most abundant unique peptide (Table 1 and *SI Appendix*, Table S2) behind actin ACTG1, which has a high frequency in MS results determined by the CRAPome database. CETN2 is a key structural component of centrosomes and regulator of centriole duplication (36). Our yeast suppressor screen suggests a role for CteG in perturbing the host cell cycle, which has centrosome-based checkpoints as centrosome defects lead to perturbations of the cell cycle (3). To validate that CteG interacts with CETN2, we immunoprecipitated FLAG-tagged CteG from infected cells transfected with HA-tagged CETN2. Only FLAG-tagged CteG pulled down HA-tagged CETN2 (Fig. 2*A*). We further confirmed these findings using an anti-CETN2 antibody to probe for an interaction with endogenous CETN2 (*SI Appendix*, Fig. S2). To determine whether this interaction is independent of other bacterial factors, we cotransfected HeLa cells with HA-tagged CETN2 and GFP-tagged CteG, TmeA, or empty vector control. Again, CteG uniquely coimmunoprecipitated with CETN2 (Fig. 2*B*). In addition, in cotransfected cells, CETN2-dsRed and GFP-tagged CteG were observed to colocalize, as evident by a significant Pearson’s R value (Fig. 2*C*). No colocalization with the negative controls GFP or TmeA-GFP was noted. To show a direct interaction between CteG and CETN2, we purified GST-tagged CETN2 and MBP-tagged CteG from *E. coli*. CteG-MBP, but not MBP alone or CpoS-MBP, bound to GST-CETN2 (Fig. 2*D*). Collectively, our results indicate that CteG specifically binds to CETN2, and that this interaction does not require any additional host or bacterial factors.

**Table 1.** AP-MS peptides of interest for CteG

Protein description	Accession	Database	Avg. score	Mass	Avg. no. of sig. matches	Avg. no. of sig. sequences
Centrin-2	P41208	UniProt_Human	844	19,726	21	9
CteG (CT105, CTL0360)	A0A654L6L6	Chlamydia_trachomatis_L2434Bu	425	68,204	11	7

### Chlamydia Amplifies Centrosomes in a CteG-Dependent Manner.

**Fig. 3.**
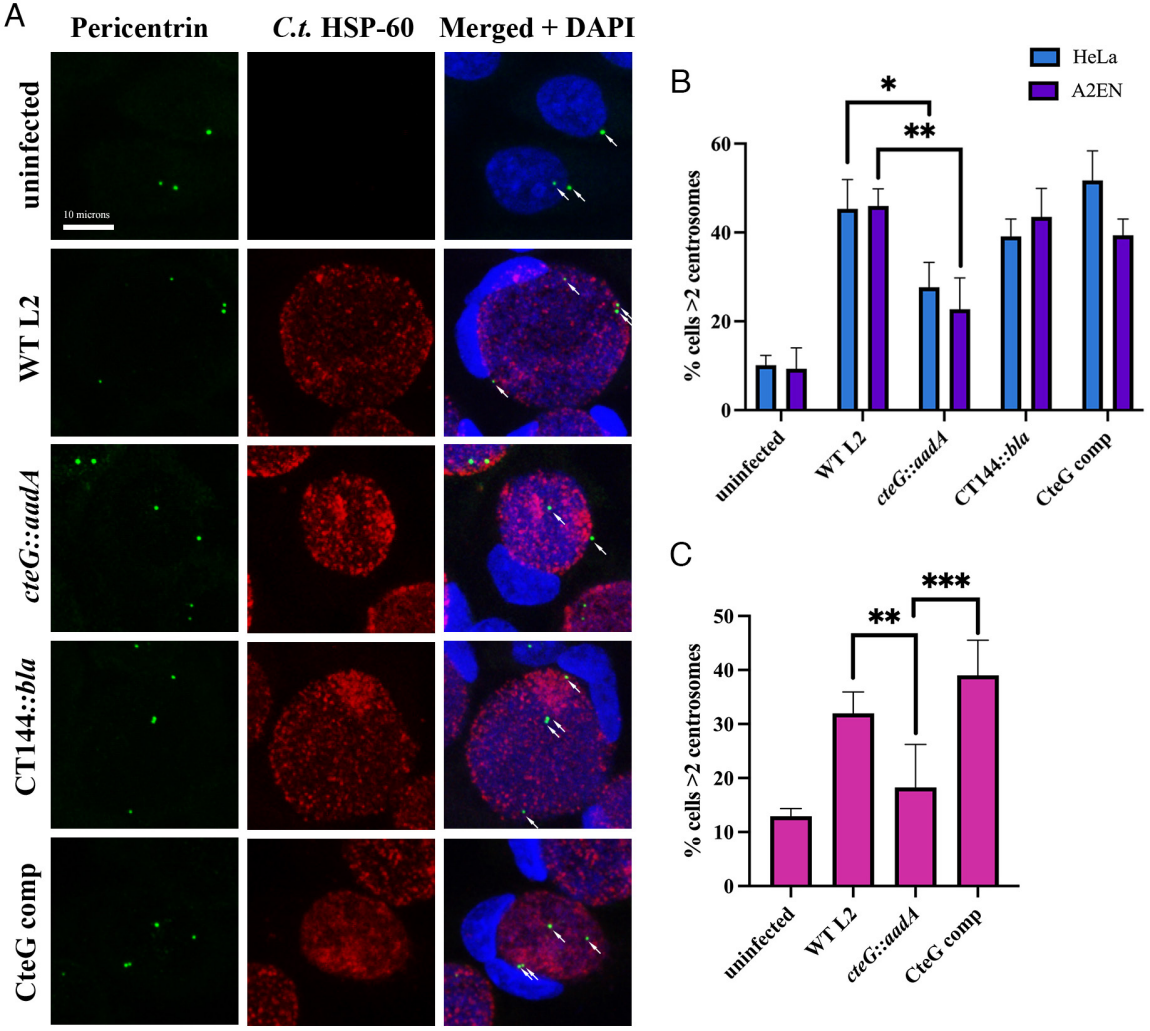
Centrosomes are amplified in a CteG-dependent manner during chlamydial infection. (*A*) Representative images of centrosomes in A2EN cells 36 h postinfection with WT L2, *cteG::aadA*, CT144*::bla*, or CteG comp. Cells were stained with *C.t.* HSP-60 (red), pericentrin (green), and DAPI (blue). White arrows indicate centrosomes (Scale bar, 10 microns). (*B*) Quantification of cells with supernumerary centrosomes (>2) at 36 h postinfection in A2EN (purple) and HeLa (blue) cells. (*C*) Quantification of cells with supernumerary centrosomes (>2) at 36 h postinfection in primary cervical cells. (*B* and *C*) Error bars are SD, *P < 0.05, **P < 0.01, ***P < 0.001. Significance was determined using one-way ANOVA followed by Tukey’s multiple comparisons test. Data are representative of two to three replicates.

It is well established that chlamydia infection can cause gross host cellular abnormalities, including centrosome amplification (18, 20, 21), but the mechanisms remain unknown. Since CteG interacts with a key structural component of the centrosome important for centriole duplication, we sought to determine whether CteG is involved in centrosome amplification during *C.t.* infection. A significant decrease in the percentage of cells with >2 centrosomes in *cteG::aadA-*infected HeLa and A2EN cells was noted compared to WT L2, CT144*::bla* (negative effector control), and CteG comp-infected cells (Fig. 3 *A* and *B*). Additionally, this same statistically significant decrease in centrosome amplification was observed between WT L2 and *cteG::aadA*-infected primary cervical cells (Fig. 3*C*). However, although not statistically significant, the presence of supernumerary centrosomes was still elevated in *cteG::aadA*-infected primary cells compared to uninfected cells, indicating that while CteG contributes to centrosome amplification during infection, it is likely not the sole contributor. Host cellular abnormalities commonly associated with *C.t.* infection, such as multinucleation and altered centrosome positioning occurred independently of CteG expression, emphasizing the specific role of CteG in centrosome amplification (*SI Appendix*, Fig. S3).

### CETN2 Is Important for Centrosome Amplification during *C.t.* Infection.

**Fig. 4.**
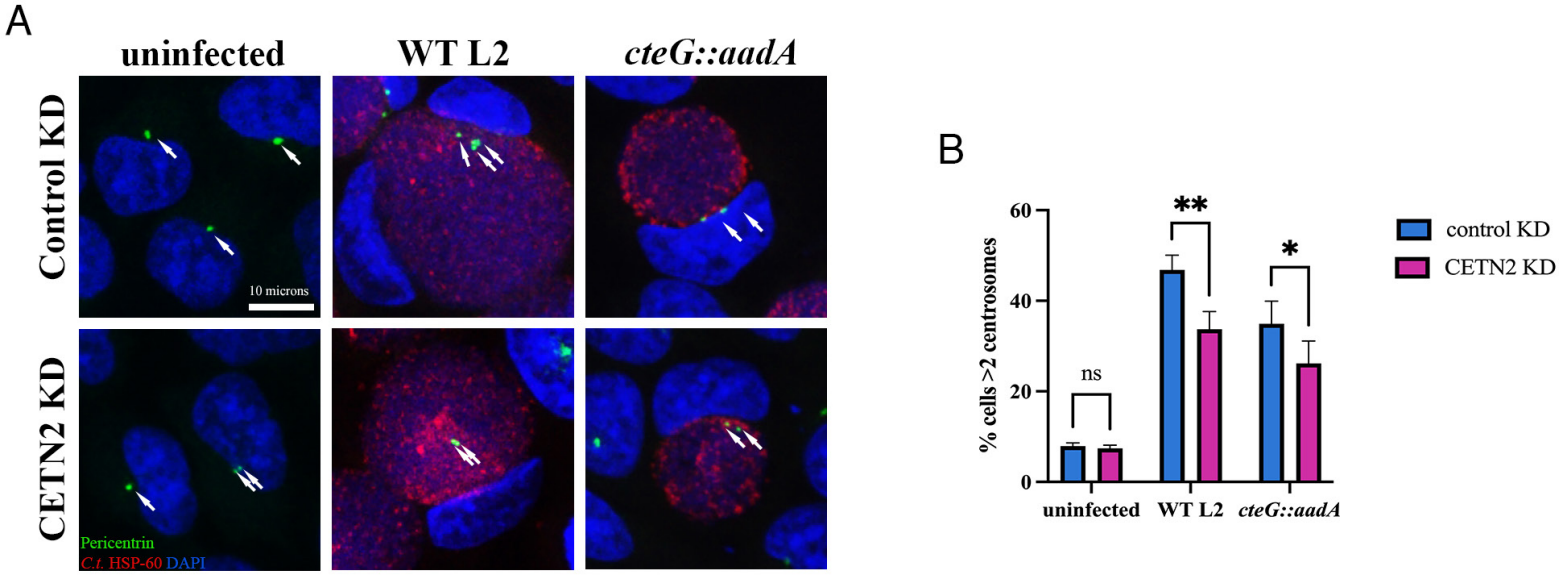
CETN2 is necessary for CteG-mediated centrosome amplification in chlamydia-infected cells. (*A*) Representative images of HeLa cells depleted (CETN2 KD, *Bottom*) or not (Control KD, *Top*) of CETN2 and infected with WT L2 or *cteG::aadA* for 36 h. Cells were stained with *C.t.* HSP-60 (red), pericentrin (green), and DAPI (blue). White arrows indicate centrosomes (Scale bar, 10 microns). (*B*) Quantification of cells with supernumerary centrosomes (>2) at 36 h postinfection in CETN2 KD (pink) and control KD (blue) cells. Error bars are SD, *P < 0.05, **P < 0.01. Significance was determined using two-way ANOVA followed Tukey’s multiple comparisons test. Data are representative of two replicates.

Our data indicate that CteG plays a role in centrosome amplification during chlamydial infection, which we hypothesize is due to its interaction with CETN2. To test this, we used siRNA to knockdown CETN2 expression. Due to low abundance of the CETN2 protein, we were unable to detect it by western blotting in cell lysates without immunoprecipitation. Thus, we used Quantigene to determine knockdown efficiency, achieving an average of a 12-fold decrease in CETN2 mRNA transcript. Knockdown of CETN2 resulted in a significant decrease in the percentage of cells with supernumerary centrosomes when infected with WT L2 or *cteG::aadA* compared to control KD (Fig. 4). Our results implicate both CteG and CETN2 in infection-associated centrosome amplification.

### The C-Terminal 39 Amino Acids of CETN2 Are Necessary for CteG Binding.

**Fig. 5.**
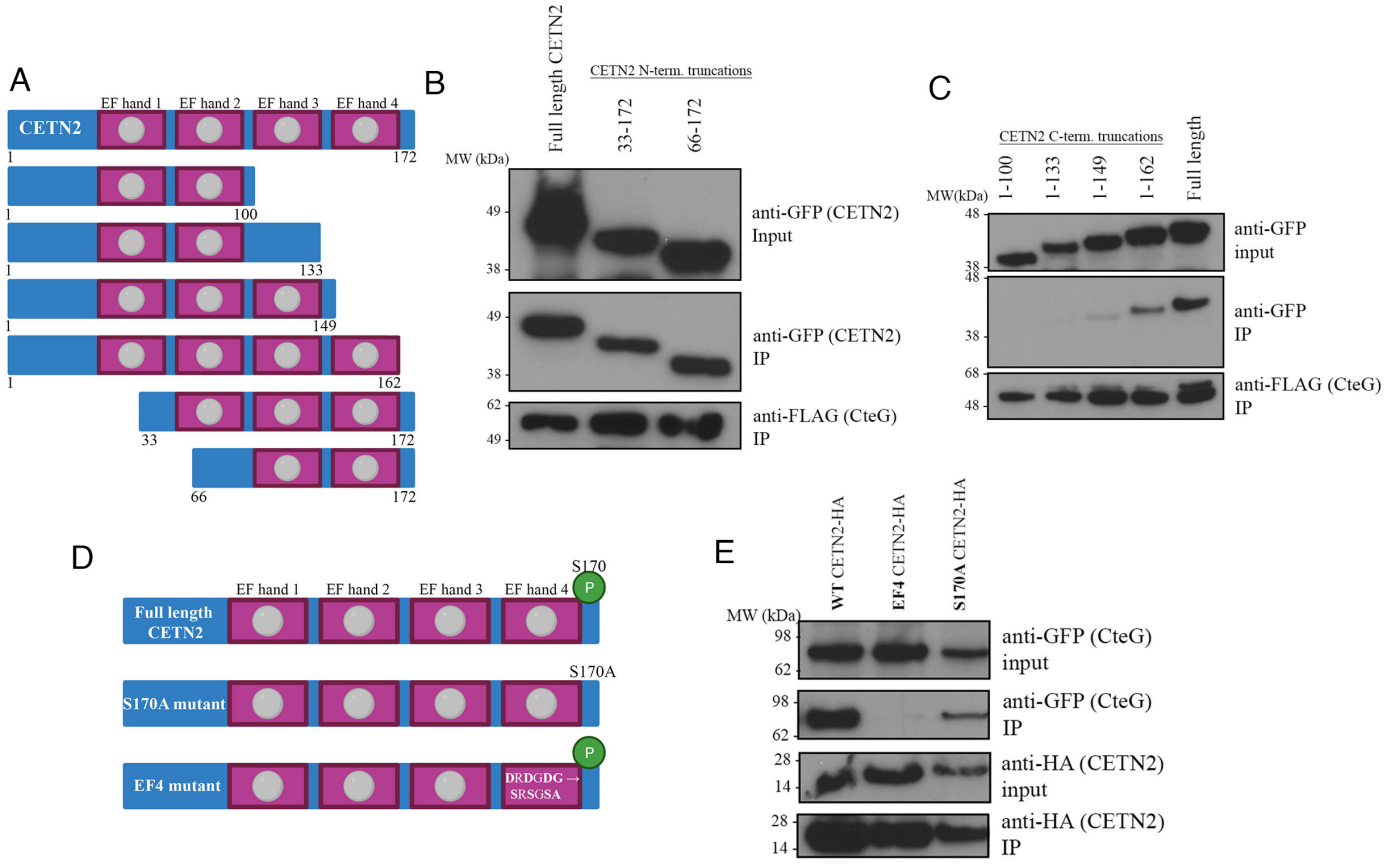
Intact C-terminus of CETN2 is needed for CteG–CETN2 interaction. (*A*) Schematic of CETN2 truncations made with full-length CETN2 at the *Top*. Pink boxes represent EF-hand domains. Gray circles indicate intact calcium-binding domains. (*B* and *C*) Co-IP of FLAG-tagged CteG from HeLa cells transfected with N- or C-terminal truncations of CETN2 and infected with *C.t.* expressing the FLAG-tagged CteG. (*D*) Schematic of CETN2 mutations made to phosphorylation site S170 and the calcium-binding domain of EF hand 4. *Top* figure shows intact calcium-binding domains and S170 phosphorylation site (green circle). (*E*) Co-IP of HA-tagged CETN2 mutants from HeLa cells cotransfected with GFP-tagged CteG and CETN2 mutants. Data are representative of two to three replicates.

CETN2 has multiple phosphorylation sites and four EF-hand domains capable of calcium binding (37). These phosphorylation sites and EF hands are important for centrin localization and formation of centrin-containing structures at the MTOC (37, 38). To determine where CteG is binding CETN2, we made sequential (~100 nucleotide/33 amino acid) truncations from the C- and N-termini of CETN2 (Fig. 5*A*). Of these, only the full length and 1-162 CETN2 readily bound to CteG with greatly reduced binding noted for 1-149 CETN2, indicating the last 39 amino acids of CETN2, which contains EF hand 4, are important for binding (Fig. 5 *B* and *C*). To determine the necessity of a viable EF hand, we made conserved mutations in the calcium-binding domain of EF hand 4 (**D**R**D**G**DG**-->**S**R**S**G**SA**) (Fig. 5*D*). This C-terminal region also contains a key phosphorylation site at serine 170, so we mutated this serine to alanine to prevent phosphorylation (denoted S170A CETN2) (Fig. 5*D*). While CteG coimmunoprecipitated with full-length (FL) CETN2, as well as S170A CETN2, no binding was noted with the EF hand 4 domain mutant (Fig. 5*E*). This highlights the importance of an intact EF hand 4 calcium-binding domain for CteG–CETN2 interaction.

### The C-Terminus of CteG Is Necessary for CETN2 Binding and Centrosome Amplification.

**Fig. 6.**
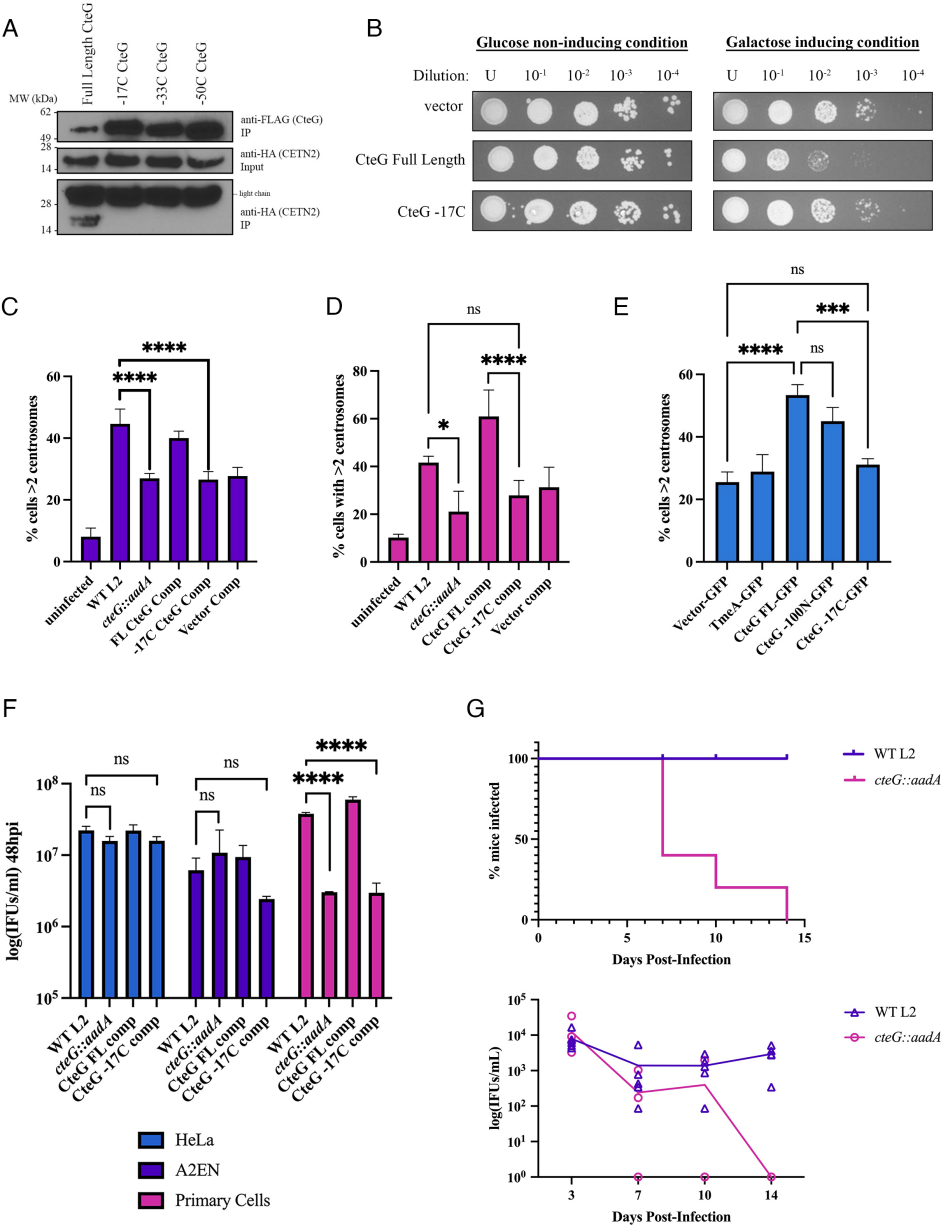
The C-terminus of CteG is necessary for CteG–CETN2 interaction, centrosome amplification, and replication. (*A*) Co-IP of FLAG-tagged CteG truncations from HeLa cells transfected with CETN2-HA and infected with *C.t.* expressing the FLAG-tagged CteG truncations. (*B*) Yeast transformed with empty vector, full-length CteG, and CteG -17C under galactose inducible promoters were diluted and spotted onto glucose- or galactose-containing media to assess toxicity. (*C–E*) Quantification of cells with supernumerary centrosomes (>2) at 36 h postinfection in A2EN cells (*C*), primary cervical cells (*D*), or HeLa cells transfected with GFP constructs and infected with *cteG::aadA* (*E*). (*F*) Quantification of infectious progenies at 48 h postinfection in HeLa (blue), A2EN (purple), and primary cervical (pink) cells normalized to WT L2 IFUs at 0 h. (*G*) C3H/HeJ mice were intravaginally infected with WT L2 or *cteG::aadA* and chlamydial shedding was monitored on days 3, 7, 10, and 14. *Top* graph shows percent of mice shedding *C.t.* at each time point. *Bottom* graph shows IFU/mL from infected mice. (*C–F*) Error bars are SD, *P < 0.05, **P < 0.01, ***P < 0.001, ****P < 0.0001. Significance was determined using one-way ANOVA followed by Tukey’s multiple comparisons test. Data are representative of two to three replicates.

To identify the regions of CteG that are necessary for this interaction, we made sequential C- and N-termini truncations and assessed binding to CETN2. While the N-terminus is dispensable (*SI Appendix*, Fig. S4), the last 17 amino acids of CteG are important for CETN2 binding (Fig. 6*A*). Importantly, the inability of the -17C CteG variant to bind CETN2 was not due to impaired secretion as no difference in secretion was noted using a GSK secretion assay (*SI Appendix*, Fig. S5*A*).

To further confirm the importance of the CteG C-terminal 17 amino acids, we again assessed toxicity in *S. cerevisiae.* Deletion of the last 17 amino acids of CteG resulted in loss of toxicity when overexpressed in yeast (Fig. 6*B*). Taken together, these experiments indicate the C-terminus of CteG is important for its function.

To determine whether the C-terminal region, and by extension binding to CETN2, is essential for centrosome amplification, we again evaluated supernumerary centrosome formation in A2EN and primary cervical cells. Expression of the FLAG-tagged FL or -17C CteG was confirmed by western blotting (*SI Appendix*, Fig. S5*B*). In A2EN cells, the -17C comp phenocopied *cteG::aadA-*infected cells and was significantly reduced compared to WT L2 (Fig. 6*C*). However, in primary cells, those infected with the -17C comp exhibited reduced supernumerary centrosomes (>2 centrosomes), but not to the degree of the *cteG::aadA* mutant (Fig. 6*D*). Interestingly, in primary cells, there was a significant decrease between CteG FL comp compared to -17C comp, suggesting this region is at least partially required for centrosome amplification.

To further evaluate this, we ectopically expressed CteG-GFP variants in *cteG::aadA*-infected cells. While CteG-GFP is unable to induce centrosome amplification in the absence of infection (*SI Appendix*, Fig. S6), it was able to restore centrosome amplification in *cteG::aadA*-infected cells (Fig. 6*E*). Furthermore, cells expressing CteG-17C-GFP were not able to restore supernumerary centrosome formation (Fig. 6*E*). Previous work showed a Golgi-localization sequence in the first 100 amino acids of the N terminus of CteG (28). As shown in Fig. 6*E*, expression of a CteG variant that lacks the first 100 amino acids of CteG does not impact centrosome amplification, indicating that Golgi targeting and centrosome amplification are two distinct features of CteG. Importantly, equal expression of the transfected constructs was noted (*SI Appendix*, Fig. S5*C*).

### CteG Is Important for Chlamydial Replication and In Vivo Infection.

As CteG is important for centrosome amplification (Fig. 3) and lytic exit (39), we next sought to determine whether CteG, and by extension supernumerary centrosomes, are important for chlamydial infection. While no growth defect was noted in HeLa or A2EN cells, a significant decrease in infectious progeny from *cteG::aadA* was noted compared to WT L2- and CteG comp-infected primary human cervical cells (Fig. 6*F*). Furthermore, the -17C comp strain also showed a significant growth defect in primary cervical cells (Fig. 6*F*). To further test the importance of CteG in chlamydia infection, we infected C3H/HeJ mice with WT L2 and *cteG::aadA* strains. Mutant-infected mice cleared infection faster than WT L2-infected mice, with all mice infected with *cteG::aadA* having cleared the infection before or by day 14 postinfection compared to zero WT-infected mice (Fig. 6*G*). Collectively these results indicate that CteG is important for normal *C.t.* replication and that this is partially due to its ability to amplify centrosomes, but likely also requires other CteG-dependent activities.

## Discussion

**Fig. 7.**
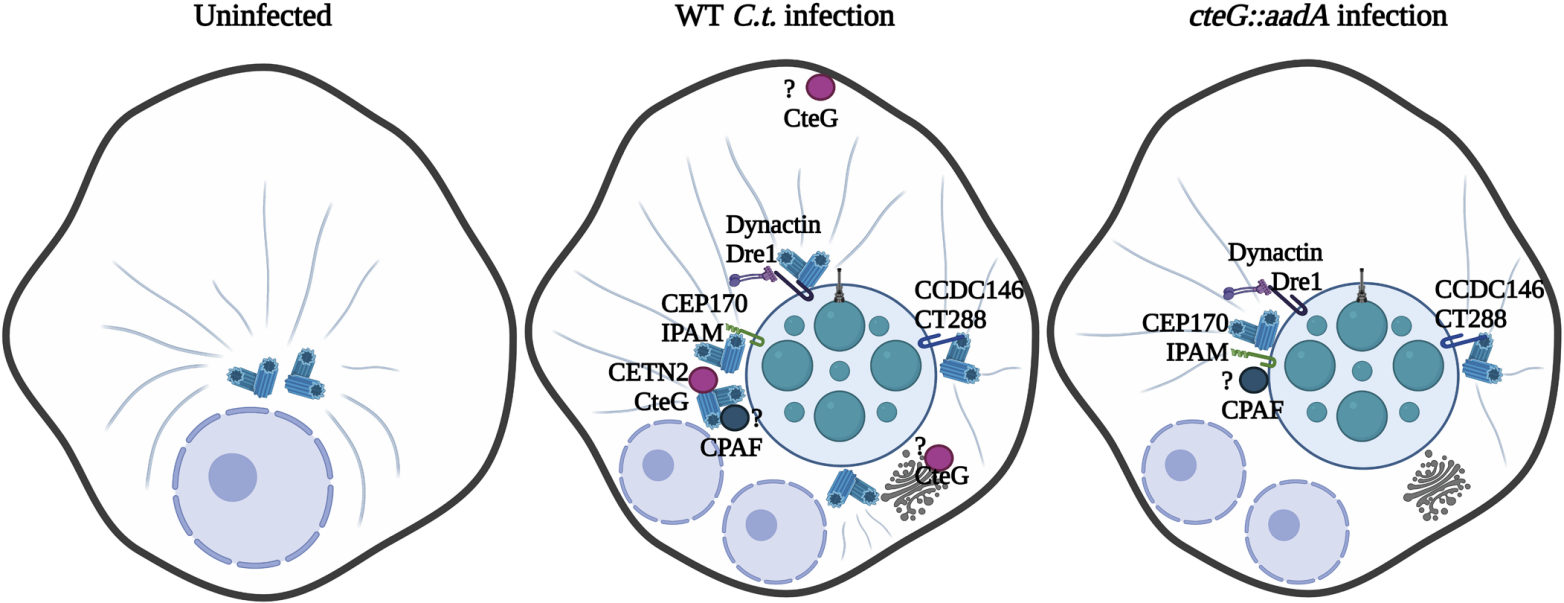
Schematic of working model. Uninfected cells maintain a normal number and localization of centrosomes. In WT L2-infected cells, CteG–CETN2 interactions promote centrosome amplification. Other effector proteins aid in anchoring the inclusion to centrosomes and positioning of centrosomes around the inclusion. Effectors are shown with known interacting partners. In *cteG::aadA*-infected cells, centrosome amplification is decreased due to the lack of CteG–CETN2 interaction, but other effectors are still present to interact with these reduced number of centrosomes.

As an obligate intracellular pathogen, *C.t.,* from the confines of its inclusion, must engage several host organelles and signaling pathways to carve out its unique replicative niche. To achieve these feats, *C.t.* releases an arsenal of T3SS effector proteins into the host cell, the function of most remains largely unknown. Our data indicate that CteG, through interactions with CETN2, contributes to induction of centrosome amplification during chlamydial infection (Fig. 7). Notably, our findings begin to dissect how a bacterial pathogen induces such cellular abnormalities as centrosome amplification that have canonically been associated with viral infections.

APC2 is a subunit of the anaphase-promoting complex/cyclosome (APC/C), an E3 ligase involved in the degradation of proteins to regulate mitotic progression. During mitosis, activation of APC/C-CDC20 only occurs once the spindle assembly checkpoint (SAC) has been fulfilled. Thus prior to anaphase, SAC prevents activation of the complex until the spindles are appropriately aligned, at which point target substrates, including cyclin B and securin, are degraded by the complex, allowing for initiation of anaphase (11). New studies have found that additionally APC/C regulates centrosome clustering, allowing cells possessing extra centrosomes to achieve bipolar mitosis and pass the SAC regulatory checkpoint (11). While CteG did not interact with APC2 directly (*SI Appendix*, Table S2), we hypothesize that CteG–CETN2 interactions induce centrosome amplification, and overexpression of APC2 would ameliorate this deleterious effect by allowing for centrosome clustering, which manifest as diminished toxicity.

Our work highlights the importance of CETN2 in the regulation of centrosome amplification in chlamydia infection. CETN2 is an important structural component of centrosomes and is a known regulator of centriole duplication (36). As a member of the EF-hand superfamily, it harbors distinct helix-loop-helix domains that coordinate calcium binding (37, 38). Binding of calcium is presumed to be important for target recognition with low-affinity sites becoming higher affinity sites in the presence of calcium (40). While CETN2 possesses four EF-hand domains, the important calcium-regulatory sites for human centrin proteins appears to be the pair at the C-terminus (41). Our data indicate that an intact calcium-binding domain of EF hand 4 is important for CteG binding (Fig. 5*E*). Given the importance of calcium binding for target recognition, we predict that calcium binding to EF hand 4 induces a conformational change that enables CteG binding. Centrosome assembly in mammalian cells requires CETN2 association with other proteins or protein complexes including CaM (calmodulin), CP110 (42), hSfi1 (43, 44), and hPOC5 (45) for appropriate centrosome duplication and mitotic spindle assembly. As many of these interactions occur at the C-terminus of CETN2, binding of CteG to this region may obscure CETN2’s interaction with other host proteins impairing regulation of the centrosome duplication process, suggesting CteG is acting as an agonist to promote centrosome amplification. Future work is needed to elucidate whether CteG perturbs CETN2 binding to its canonical binding partners.

Our findings add to the growing body of literature that link *C.t.* infection to induction of host cellular abnormalities, such as supernumerary centrosomes, mitotic spindle defects, multinucleation, aneuploidy, and blocked cytokinesis that were initially described over 15 y ago, but are still mechanistically undefined (18, 20–24). To date, most studies have been performed in HeLa cells or E6/E7 transformed cell lines, clouding whether observed phenotypes are due to *C.t.* infection or are artifacts of HPV infection in these cell lines. Recent work by Wang et al. showed that centrosome amplification is an additive effect between HPV and *C.t*. (20), but this occurs through different mechanisms. Using HPV-negative cell lines, they show that centrosome amplification requires progression through the cell cycle and may result from a cytokinesis defect. Building on these findings, our data indicate that centrosome amplification can also be induced through CteG–CETN2 interactions. Intriguingly, HeLa or A2EN cells infected with *cteG::aadA* have significantly reduced centrosomes relative to cells infected with WT L2, yet the number of centrosomes present in the *cteG::aadA*-infected cells are still elevated relative to uninfected cells (Fig. 3*B*). In primary cells, the percent of *cteG::aadA*-infected cells with supernumerary centrosomes mirrored that of uninfected cells (Fig. 3*C*). Interestingly, both the *cteG::aadA* and the -17C comp were impaired in intracellular replication in primary cells (Fig. 6*F*). One possible model to explain these data is that the growth defect is directly due to its inability to amplify centrosomes. Immortalized cells such as A2EN and HeLa express oncogenes that promote centrosome amplification. Thus, extra centrosomes in these cells may still provide a permissive environment without CteG and may help explain why the CteG mutant is only impaired in growth in primary cells and in vivo. Alternatively, CteG has also been attributed to carrying out functions at the Golgi and plasma membrane and is involved in lytic exit of chlamydia (39). Given the plethora of activities associated with this secreted factor, more work is needed to precisely define why CteG is necessary for chlamydial infection.

While CteG appears to be necessary for centrosome amplification, it is not sufficient, highlighting the importance of other chlamydial factors in this process. A recent study by Sherry et al. revealed that the inclusion membrane protein, Dre1, interacts with dynactin to reposition host organelles, namely centrosomes, to help with the positioning of the *C.t.* inclusion at the MTOC (46). Dre1 is responsible for overriding normal host centrosome clustering mechanisms to allow *C.t.* to position centrosomes in close proximity to the inclusion. Other Incs, including CT223/IPAM and CT288 bind to centrosome components (47, 48). IPAM has been associated with centrosome amplification and failed cytokinesis in IPAM-transfected cells (25). IPAM also recruits CEP170, a centrosomal protein, to control microtubule organization and assembly from the inclusion. CT288 was shown to interact with human centrosomal protein CCDC146 and is partially responsible for recruiting it to the inclusion membrane during infection, potentially playing a role in inclusion anchoring at the MTOC (48). Collectively, these studies support a role for Inc proteins in the positioning of the inclusion at the MTOC through repositioning of centrosomes. Previous studies also suggest that the secreted factor CPAF may be important for centrosome amplification (23). As ectopic expression of CteG is insufficient to induce centrosome amplification in the absence of infection, other bacterial factors are required, and it is intriguing to speculate it may be one of the aforementioned Incs or CPAF. Taken together, our results, in conjunction with these prior studies, suggest that chlamydia employs multiple secreted factors that induce supernumerary centrosome formation, potentially to aid in positioning the inclusion at the MTOC.

Previous work on CteG showed localization to the Golgi or plasma membrane depending on the stage of the infection (28). More recent work implicated CteG in *C.t.* lytic exit from the host (39). This study found decreased host cell cytotoxicity in *cteG*::*bla*-infected cells, indicating a role for this effector in host cell lysis at the end of the *C.t.* lifecycle to facilitate release of infectious chlamydia (39). Previous work showed the first 100 amino acids of CteG to be necessary for Golgi localization. Herein, we show this region is not important for centrosome amplification (Fig. 6*E*), nor did our AP-MS yield any Golgi proteins (*SI Appendix*, Table S2). The Golgi and centrosomes are adjacent during interphase and there are implications for the Golgi functioning to regulate directional protein transport, centrosome positioning, centrosome morphology, and cell cycle progression (49). While our results highlight CteG in perturbing normal centrosome duplication, they do not exclude the possibility that CteG is a multifunctional protein that may also directly interact with Golgi proteins. Future work should include identifying binding partners at more timepoints postinfection than just 24 h to assess the multifunctional rules of CteG. Additionally, centrosomes are less clustered in *C.t.-*infected cells (46), so CteG may be localizing with these centrosomes around the mature inclusion at the plasma membrane, where it could then help facilitate lytic exit later in the infection cycle. As centrosomes serve as important microbial tracks, it is possible that less microtubules encompass the inclusion in a CteG mutant strain, which leads to changes in lytic exit.

Taken together, we propose a model where upon infection, CteG is secreted and traffics to the Golgi apparatus/MTOC and plasma membrane. Through interactions with CETN2, CteG induces centrosome amplification in a manner that requires additional bacterial factors. We posit centrosome amplification may serve to aid in the positioning of the inclusion at the MTOC through interactions with inclusion membrane proteins (Fig. 7). We speculate that changes in cell cycle progression (as measured by EdU staining) are a downstream effect of CteG’s primary effect on centrosome amplification, as this amplification process likely alters cell cycle progression, and centrosome duplication is heavily linked to cell cycle progression. Further characterization of the CteG–CETN2 interaction is necessary to understand the mechanistic underpinnings of this interaction and how it leads to centrosome amplification. This would contribute to our understanding of how *C.t.* induces gross host cell abnormalities that are also hallmarks of cancer, potentially providing a link between *C.t.* infection and increased cancer risk.

## Materials and Methods

### Bacterial and Cell Culture.

*C.t.* serovar L2 (LGV 434/Bu) was propagated in HeLa 229 cells (American Type Tissue Culture), and EBs were purified using a gastrograffin density gradient as previously described (50). HeLa cells were grown in RPMI 1640 with L-Glutamine (Thermo Fisher Scientific) supplemented with 10% Fetal Bovine Serum (Gibco), sodium bicarbonate, sodium pyruvate, and gentamicin at 37 °C with 5% CO_2_. A2EN cells (Kerafast) were propagated in keratinocyte-serum free media (K-SFM) (Thermo Fisher Scientific) supplemented with 0.16 ng/mL epidermal growth factor, 25 μg/mL bovine pituitary extract, 0.4 mM CaCl_2_, and gentamicin (51, 52). Primary cervical cells were derived from normal HPV-negative cervical tissue obtained through the University of Iowa Tissue Procurement Core from a consented donor who underwent a hysterectomy for endometriosis (IRB#201103721 and IRB#199910006). Normal cervical epithelial cells were isolated as previously described (53) and were maintained in K-SFM (Thermo Fisher Scientific) without CaCl_2_.

### Cloning.

TargeTronics was used to predict TargeTron insertion sites for CT144 and gBlock fragments were obtained from Integrated DNA Technologies (*SI Appendix*, Table S1). These gBlocks were cloned into the HindIII/BsrGI site of pACT (54). For secretion validation or complementation, CteG FL or CteG -17C were expressed as a C-terminal fusion to GSK or FLAG tag by cloning the fusion protein into the NotI/KpnI site of pBomb4. GFP constructs were cloned into pcDNA3.1-N-GFP at either the KpnI/XhoI, KpnI/XbaI, or KpnI/NotI sites. For purifying proteins, CteG and CpoS were cloned into pMAL-c5VT using NotI/SalI sites and CETN2 was cloned into pGEX-6P-1 using NotI/SalI sites.

### Chlamydia Transformation.

*C.t.* EBs were transformed as previously described (54) with minor modifications. Briefly, fresh *C.t.* lysates of WT L2 for FLAG-tagged CteG truncation constructs or *cteG::aadA* for complementation with FLAG-tagged CteG were mixed with 5 µg plasmid DNA and 10 µL 5X transformation mix (50 mM Tris pH 7.4 and 250 mM CaCl_2_) in a total volume of 50 µL. Mixtures were incubated at room temperature for 30 min, resuspended in RPMI, and applied to 2 wells of a 6-well plate of confluent HeLa cells. Plates were centrifuged at 900 × g for 30 min. At 18 h postinfection, 0.3 µg/mL penicillin G was added. Infectious progenies were harvested every 48 h and used to infect a new HeLa cell monolayer until viable inclusions were present (~2 to 3 passages). Expression of FLAG-tagged proteins was confirmed by western blotting. For TargeTron mutants, successful insertion into the target gene was confirmed by PCR.

### Yeast Suppressor Screen.

To identify putative suppressors of CteG toxicity in yeast, a yeast suppressor screen was carried out as previously described (30). Briefly, CteG was cloned into the KpnI/XbaI site of pYesNTA and the resulting plasmid (pYesNTA-CteG) was transformed into *S. cerevisiae* W303. To assess toxicity, transformants were serially diluted and spotted onto uracil dropout medium containing glucose or galactose as the sole carbon source. To identify yeast ORFs that suppress CteG toxicity, the pYEp13 genomic library (ATCC no. 37323) was transformed into the W303-CteG strain. Transformants were plated on uracil leucine dropout medium containing galactose. From a total transformation of ~1.0 × 10^5^, we obtained a total of 69 colonies. Plasmids were isolated from clones that consistently suppressed the toxicity of CteG, and isolated plasmids were retransformed into W303-CteG to confirm suppression. To identify yeast ORFs present, suppressor plasmids were sequenced using pYEp13 seq F and pYEp13 seq R (*SI Appendix*, Table S1). Sequences were analyzed using the yeast genome database (https://www.yeastgenome.org/). To validate suppression, putative suppressors were then individually cloned into p415-ADH (31).

### Affinity Purification.

HeLa cells were infected at an MOI of 2 with *C.t.* strains expressing a FLAG-tagged effector protein, under tetracycline inducing conditions (10 ng/mL) for 24 h. Cells were lysed in eukaryotic lysis solution (ELS) (50 mM Tris HCl, pH 7.4, 150 mM NaCl, 1 mM EDTA, and 1% Triton-X 100) and spun at 12,000 × g for 20 min. Supernatants were incubated with 60 µL preclearing beads (mouse IgG agarose, Millipore Sigma) for 2 h. The precleared lysate was incubated with 30 µL FLAG beads (anti-FLAG M2 Affinity Gel, Millipore Sigma) overnight. The beads were washed 6 times with ELS without detergent. For mass spectrometry, samples were stored in 50 mM ammonium bicarbonate prior to digestion and analysis. For western blotting, proteins were eluted from the beads in NuPAGE LDS Sample Buffer (Thermo Fisher Scientific) and boiled for 5 min.

### Mass Spectrometry.

Beads containing samples were washed with 25 mM ammonium bicarbonate and digested with 0.5 μg trypsin (Pierce, Thermo Fisher Scientific, MS Grade) using a CEM microwave reactor for 30 min at 55 °C. Digested peptides were extracted twice using 50% acetonitrile plus 5% formic acid, lyophilized to dry, and resuspended in 5% acetonitrile plus 0.1% formic acid. For LC/MS, samples were injected into an UltiMate 3000 UHPLC system coupled online to a high-resolution Thermo Orbitrap Fusion Tribrid mass spectrometer. Peptides were separated by reversed-phase chromatography using a 25-cm Acclaim PepMap 100 C18 column with mobile phases of 0.1% formic acid and 0.1% formic acid in acetonitrile; a linear gradient from 4% formic acid in acetonitrile to 35% formic acid in acetonitrile over the course of 45 min was employed for peptide separation. The mass spectrometer was operated in a data-dependent manner, in which precursor scans from 300 to 1,500 m/z (120,000 resolution) were followed by collision-induced dissociation of the most abundant precursors over a maximum cycle time of 3 s (35% NCE, 1.6 m/z isolation window, 60-s dynamic exclusion window). Raw LC-MS/MS data were searched against a database containing UniProt_Human and Chlamydia_trachomatis_L2434Bu using Mascot 2.8. Tryptic digestion was specified with a maximum of two missed cleavages, while peptide and fragment mass tolerances were set to 10 ppm and 0.6, respectively. Quantitation was done using Mascot Average method using Mascot Distiller 2.8.2.

### Coimmunoprecipitations.

Coimmunoprecipitations were performed on either cotransfected HeLa cells or cells that were transfected using Lipofectamine LTX (Thermo Fisher Scientific) and subsequently infected at an MOI of 2.5 for 24 h. Cells were lysed with ELS and spun at 12,000 × g for 20 min. Supernatants were incubated with 50 µL FLAG magnetic beads (Pierce™ Anti-DYKDDDDK, Thermo Fisher Scientific) for 2 h. The beads were washed 6 times with ELS without detergent. Proteins were eluted from the beads in NuPAGE LDS Sample Buffer (Thermo Fisher Scientific) and boiled for 5 min prior to analysis by western blotting.

### Western Blotting.

Samples were separated by Sodium dodecyl-sulfate polyacrylamide gel electrophoresis (SDS-PAGE) and transferred to PVDF membranes. Blots were blocked in 5% milk in Tris-buffered saline with Tween 20. Membranes were probed with an anti-GFP (Novus), anti-FLAG (Thermo Fisher Scientific), or anti-HA (Millipore Sigma) primary antibody and goat anti-rabbit HRP conjugate (BioRad) secondary antibody. To evaluate secretion, anti-GSK or anti-P-GSK (Cell Signaling) antibodies were used. Results were collected from at least three independent experiments.

### Immunofluorescence.

HeLa cells were cotransfected with CETN2-dsRed and GFP-tagged *C.t.* effectors CteG, TmeA, or empty vector. Cells were fixed in 4% formaldehyde, permeabilized with 0.1% Triton-X and stained with DAPI. Images were taken on a Nikon Eclipse Ti2 microscope. Images were analyzed for colocalization using ImageJ Coloc2 function to calculate a Pearson’s R value. Values greater than 0.7 are considered significant.

### Protein Purification.

Protein purification and pulldowns were conducted as previously described (55) with minor modifications. Expression of GST-CETN2, MBP, MBP-CT105, or MBP-CT229 was induced overnight by the addition of isopropyl-β-thiogalactopyranoside (1 mM, final concentration) to a 1 L culture *E. coli* BL21-λDE3 at OD 0.8. Bacterial pellets were stored at −80 °C. Pellets were resuspended in 100 mL sonication buffer (For GST: 50 mM Tris pH 7.5, 500 mM NaCl, 10% glycerol and 1 mM DTT; for MBP: 20 mM Tris pH 7.5, 200 mM NaCl, 1 mM EDTA, 1 mM Sodium Azide, 10% glycerol, and 1 mM DTT) and sonicated using six 30-s pulses at 80% power and then centrifuged at 7,500 rpm for 10 min at 4 °C. Then, 500 μL glutathione agarose beads for GST-tagged constructs and 500 μL Amylose resin for MBP-tagged constructs were added to a gravity filtration column and washed three times with sonication buffer. Cleared lysate was run-through the column, and then the beads washed three more times with sonication buffer. Successful purification was confirmed using Coomassie staining. MBP, MBP-CT105, and MBP-CT229 were eluted from amylose resin using 2 mL MBP sonication buffer supplemented with 10 mM D-(+)-maltose monohydrate. GST-CETN2 attached to glutathione beads was washed three times in sonication buffer. Then, 2 mL sonication buffer containing 20 μg MBP, MBP-CT105, or MBP-CT229 was added to each tube with GST beads, and binding was allowed to occur overnight at 4 °C with rotation. Following overnight binding, beads were washed three times in wash buffer (20 mM Tris pH 7.5, 300 mM NaCl, 1 mM EDTA, 1 mM MgCl_2_, and 1% Triton-x-100). After the final wash, proteins were eluted from glutathione agarose resin using 2 mL GST sonication buffer supplemented with 50 mM Tris pH 7.5 150 mM NaCl and 1 mM DTT. Samples were eluted from the beads in NuPAGE LDS Sample Buffer (Thermo Fisher Scientific) and boiled for 5 min prior to analysis by western blotting probing with anti-GST or anti-MBP antibodies.

### Centrosome Staining.

Immunofluorescence centrosome staining was done as previously established with modification (20, 46). HeLa, A2EN, or primary cervical cells were infected with the appropriate strains of *C.t.* at an MOI of 1 by centrifugation at 700 × g for 30 min. At 36 h postinfection, cells were fixed on ice with cold methanol for 6 min and blocked for 2 h at room temperature in 0.1% Triton-X in PBS with 2% FBS. Cells were stained with anti-pericentrin (abcam) and anti-Chlamydia HSP60 (Millipore Sigma). Dylight-488 and Dylight-594 (Thermo Fisher Scientific) secondaries were used along with DAPI (Thermo Fisher Scientific) to stain the nuclei. Images were captured using a Leica DFC7000T confocal microscope equipped with Leica software. At least 10 images were collected per coverslip, with three technical replicates per biological replicate, with at least 2 biological replicates.

### Centrosome Measurements.

For centrosome number measurements, maximal projection images obtained from confocal imagery were used for counting the number of centrosomes per cell. Cells with >2 centrosomes were considered to have “supernumerary centrosomes.” All centrosomes of infected cells from at least 10 images per technical replicates were counted, with at least 2 biological replicates per cell type. To measure centrosome clustering, ImageJ was used to create a polygon encompassing all centrosomes in a cell and the area of this shape measured. To measure centrosome spread, the distance from each centrosome to the nearest edge of the nucleus was determined in ImageJ. Centrosomes on the nucleus were given a value of zero. A total of 100 measurements were taken for each condition. For infected conditions, only *C.t.-*infected cells were analyzed.

### Edu Labeling.

Confluent HeLa cell monolayers were infected with the appropriate strains of *C.t.* at an MOI of 1 by centrifugation at 700 × g for 30 min. At 36 h postinfection, cells were incubated with 10 μM EdU for 30 min at 37 °C using the Click-iT EdU Cell Proliferation kit (Thermo Fisher Scientific, C10337). Samples were then fixed with 4% formaldehyde and permeabilized with 0.5% Triton-X. At least 10 images were collected (by Nikon Eclipse Ti2 microscope) per coverslip, with three technical replicates per biological replicate, with at least 2 biological replicates.

### Growth Curve.

HeLa cells were infected at an MOI 2.5 on ice. After 30 min, media were changed, and plates were moved to 37 °C with 5% CO_2_ to stimulate bacterial uptake. At 0 or 48 h, cells were lysed in water, and lysates were used to infect fresh monolayers of HeLa cells. Titer plates were fixed with MeOH 24 h after infection and stained with anti-chlamydial LPS (Novus). Infectious forming units (IFUs) were determined from counting 10 fields of triplicate samples. IFUs at 48 h were normalized to the WT L2 IFUs at 0 h.

### Intravaginal Infection of Mice.

The animal study (protocol #8112197) was reviewed and approved by the Institutional Animal Care and Use Committee, University of Iowa. Female 6 to 8-wk-old C3H/HeJ mice (The Jackson Laboratory) were pretreated with 2.5 mg medroxyprogesterone acetate at 3 and 10 d prior to infection as previously described (56). Five mice per group were intravaginally infected with 5 × 10^5^ Infectious forming units of WT or *cteG::aadA*. Chlamydial shedding was monitored on days 3, 7, 10, and 14. Recovered IFUs were enumerated by plating on HeLa cell monolayers.

### siRNA Knockdown.

HeLa cells were transfected using Dharmafect with SmartPool siRNA for CETN2 or ON-TARGET*plus* Cyclophilin B control according to the manufacturer’s protocol (Dharmacon). At 36 h posttransfection, cells were infected with the appropriate strains of *C.t.* at an MOI of 1 by centrifugation at 700 × g for 30 min and incubated at 37 °C with 5% CO_2_ for 36 h. Cells were then fixed and stained for centrosomes as described above. Knockdown efficiency was determined using QuantiGene™ (Thermo Fisher Scientific) according to the manufacturer’s protocol.

### Statistics.

When necessary, statistical analysis was performed using GraphPad Prism 9.3.0 software. One-way and two-way ANOVAs were used followed by Tukey’s multiple comparisons with *P* < 0.05 (*), *P* < 0.01 (**), and *P* < 0.001 (***).

## Supplementary Material

Appendix 01 (PDF)

## Data Availability

All study data are included in the article and/or *SI Appendix*.
