# Supplementary material for: The Chlamydia trachomatis type III-secreted effector protein CteG induces centrosome amplification through interactions with centrin-2
Source: Proc Natl Acad Sci U S A. 2023 May 8;120(20):e2303487120. doi: 10.1073/pnas.2303487120 (PMC10193975; doi:10.1073/pnas.2303487120)
Supplement: Supplementary file 1 — Appendix 01 (PDF) [file pnas.2303487120.sapp.pdf]

SUPPORTING INFORMATION

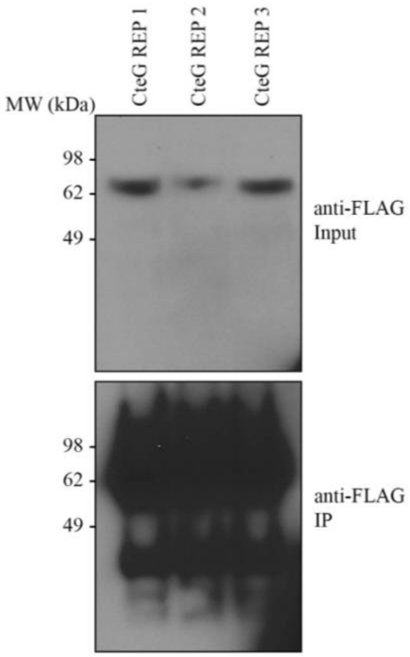

Fig. S1. Check of CteG-FLAG expression for AP-MS samples. IP of FLAG-tagged CteG from HeLa cells infected with *C.t.* expressing FLAG-tagged CteG. A portion of the three replicates sent for mass spectrometry.

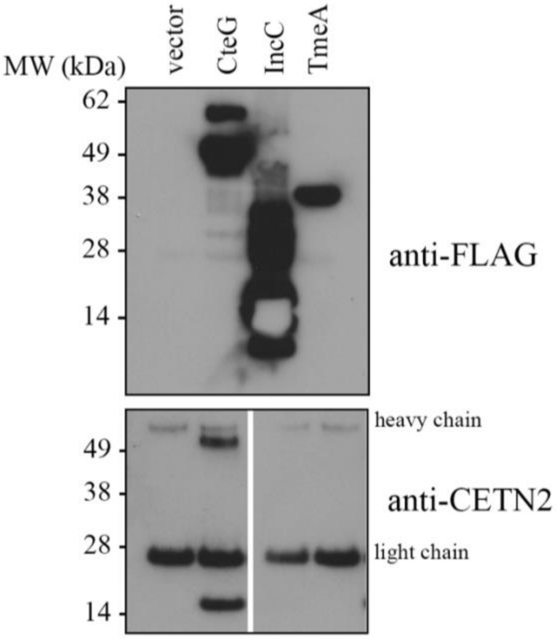

Fig. S2. CteG co-IPs with endogenous CETN2. Co-IP of FLAG-tagged effectors from HeLa cells infected with *C.t.* expressing the FLAG-tagged vector, CteG, IncC, or TmeA. Blot probed with antibody against CETN2 to look for endogenous interaction with *C.t.* effectors. CETN2 is ~19kDa.

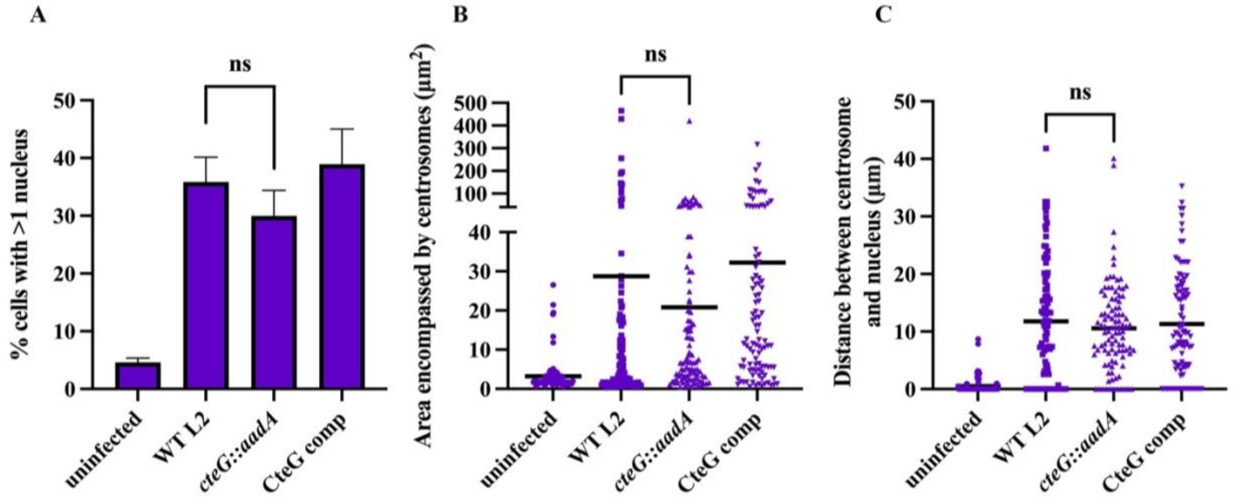

Fig. S3. CteG does not affect multinucleation, centrosome clustering, or centrosome distribution. (*A*) Quantification of cells with multinucleation (>1) at 36 hours post infection in A2EN cells represented as percent of total cells. Error bars are SD. Data are representative of 2 replicates. (*B*) Measurement of the area encompasses by centrosomes in A2EN cells. Black bars represent the mean. Data are representative of 100 counted cells. (*C*) Measurement of the distance between centrosomes to the nearest edge of the nucleus in A2EN cells. Black bars represent the mean. Data are representative of 100 counted cells. (*A*-*C*) Significance was determined using one-way ANOVA followed by Tukey's multiple comparisons test.

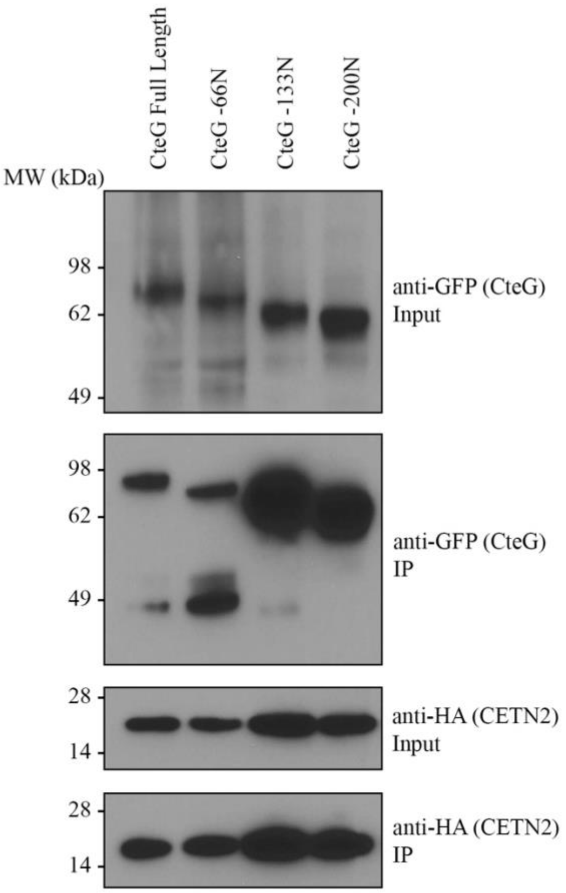

Fig. S4. N-terminus is dispensable for CteG interaction with CETN2. Co-IP of HA-tagged CETN2 from HeLa cells co-transfected with GFP-tagged CteG truncations and CETN2-HA.

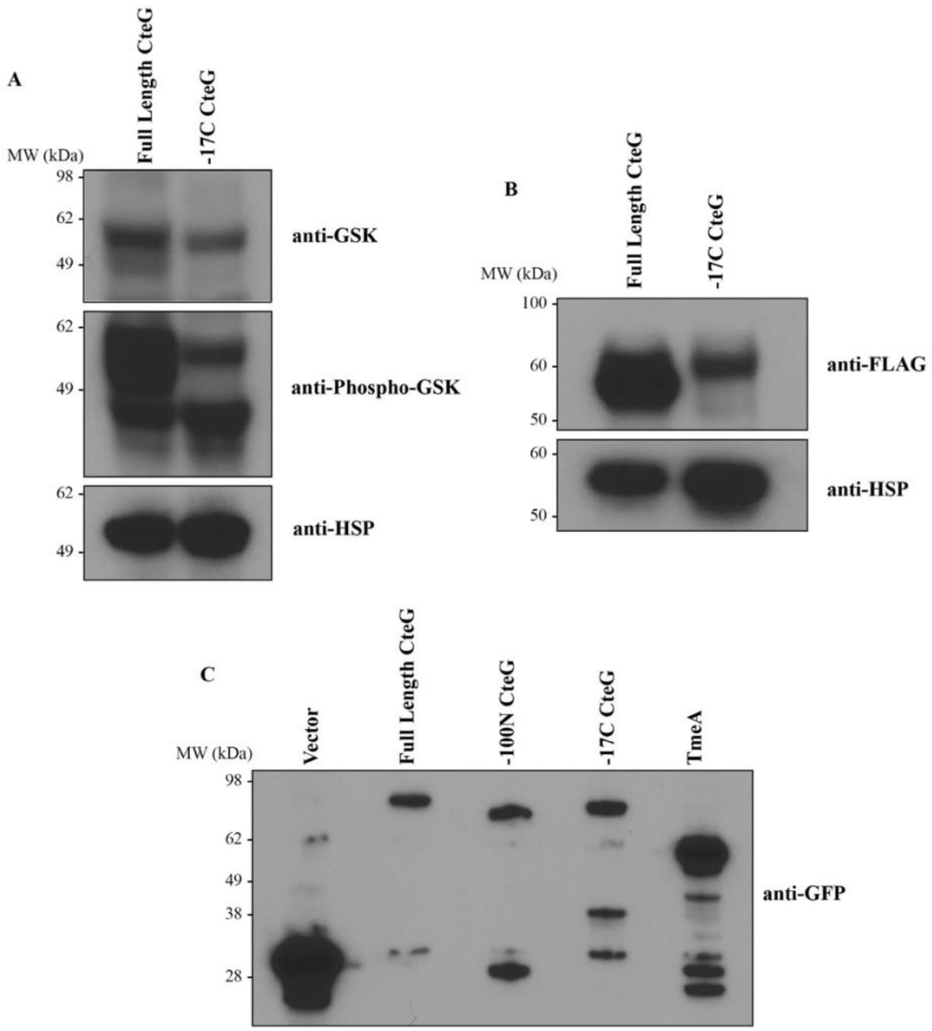

Fig. S5. CteG Full Length (FL) and -17C are secreted and expressed similarly in infection and by transfection. (*A*) Lysates from HeLa cells infected with FL or -17C GSK-tagged CteG expressing *C.t.* were probed with anti-GSK or Phospho-GSK antibodies to look at expression and secretion, respectively. Anti-HSP used as a loading control. (*B*) Lysates from HeLa cells infected with FL or -17C FLAG-tagged CteG expressing *C.t.* were probed with anti-FLAG to look at expression levels. Anti-HSP used as a loading control. (*C*) Lysates from HeLa cells transfected with GFP-tagged CteG constructs, empty vector, or TmeA. were probed with anti-GFP to assess expression levels.

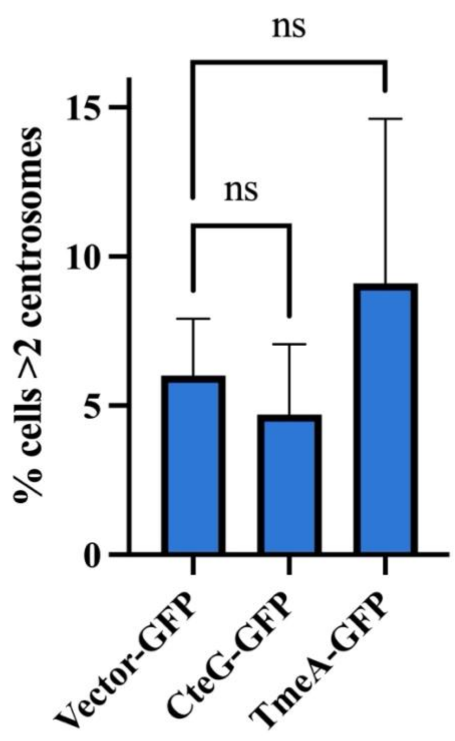

Fig. S6. CteG is not sufficient to induce centrosome amplification. Quantification of cells with supernumerary centrosomes (>2) at 36 hours post transfection in HeLa cells transfected with GFP-tagged empty vector, CteG, or TmeA.

Table S1. Primers used in this study.

Name	Use	Sequence
CT144 33/34s	TargeTron mutagenesis	TTCCCCTCTAGAAAAAAGCTTATAATTATCCTTAACTATCGATGTTGTGCGCCCAGATAGGGTGTTAAGTCAAGTAGTTTAAGGTACTACTCTGTAAGATAACACAGAAAACAGCCAACCTAACCGAAAAGCGAAAGCTGATACGGGAACAGAGCACGGTTGGAAAGCGATGAGTTACCTAAAGACAATCGGGTACGACTGAGTCGCAATGTTAATCAGATATAAGGTATAAGTTGTGTTTACTGAACGCAAGTTTCTAATTTCGATTATAGTTCGATAGAGGAAAGTGTCTGAAACCTCTAGTACAAAGAAAGGTAAGTTAGAAACATCGACTTATCTGTTATCACCACATTTGTACAATCTG
LtrB F	TargeTron mutagenesis	TTCCCCTCTAGAAAAAAGCTTATAATTATCCTTA
LtrB R	TargeTron mutagenesis	CAGATTGTACAAATGTGGTGATAACAGATAAGTC
pYEp13 sequencing F	Yeast suppressor screen sequencing	ACTACGCGATCATGGCGA
pYEp13 sequencing R	Yeast suppressor screen sequencing	TGATGCCGGCCACGATGC
APC2 HindIII F	Yeast suppressor screen	CCAAGCTTATGTCATTTCAGATTACCCCAAC
APC2 SalI R	Yeast suppressor screen	CCGTCGACTCATGAGTTTTTATGCCCATTTT
CETN2 KpnI F	CETN2 truncations	CCGGTACCATGGCCTCCAACTTTAAGAAGGC
CETN2 XhoI R	CETN2 truncations	CCCTCGAGTTAATAGAGGCTGGTCTTTTTCAT
CETN2 1-133 XhoI R	CETN2 truncations	CCCTCGAGTTACCAACTCCTTGGCCACGC
CETN2 1-100 XhoI R	CETN2 truncations	CCCTCGAGTTATTTCTCAGACATTTTCTGGGTCA
CETN2 1-149 XhoI R	CETN2 truncations	CCCTCGAGTTAAGCTTCATCAATCATTTCCTGC
CETN2 1-162 XhoI R	CETN2 truncations	CCCTCGAGTTAGAACTCTTGCTCACTGACCTCTC
CETN2 33-172 KpnI F	CETN2 truncations	CCGGTACCCGGGAAGCTTTTGATCTTTTCG
CETN2 66-172 KpnI F	CETN2 truncations	CCGGTACCGAAGAAATTAAGAAAATGATAAG
CETN2 NotI F	protein purification cloning	CCGCGGCCGCATGGCCTCCAACTTTAAGAAGGC
CETN2 SalI R	protein purification cloning	CCGTCGACATAGAGGCTGGTCTTTTTC
CteG +1 KpnI F	Yeast suppressor screen	CCGGTACCAATGTCATTTGGTATTGGTAGTGCTT
CteG Xba1 R	Yeast suppressor screen/Ectopic expression	CCTCTAGACTAGATAGAGGAGCTTTGCACAC
CteG KpnI F	Ectopic Expression	CCGGTACCATGTCATTTGGTATTGGTAGTGC
CteG NotI F	CteG truncations/ pBomb4 plasmid cloning, GSK cloning, protein purification cloning	CCGCGGCCGCATGTCATTTGGTATTGGTAGTGCTT

CteG FLAG KpnI R	CteG truncations, GSK cloning	CCGGTACCTTACTTATCGTCGTCATCCTTGTAATC GATAGAGGAGCTTTGCACACCT
CteG FLAG SalI R	CteG truncations/ pBomb4 plasmid cloning	CCGTCGACTTACTTATCGTCGTCATCCTTGTAATC GATAGAGGAGCTTTGCACACCT
CteG SalI R	protein purification cloning	CCGTCGACCTAGATAGAGGAGCTTTGCACA
CteG -100N KpnI F	CteG truncations	CCGGTACCGGAGTAAGTCTTACATCTATATC
CteG -66N KpnI F	CteG truncations	CCGGTACCCCAATGGTAGGGACGTACTCAG
CteG -133N KpnI F	CteG truncations	CCGGTACCTCTGCAAGAGGTGCTGGTTCC
CteG -200N KpnI F	CteG truncations	CCGGTACCTTTCTTGCTTTAGGAGGAT
CteG -66C Xba1 R	CteG truncations	CCTCTAGAGGATGATTGCAAATGCGTTAAAC
CteG -133C Xba1 R	CteG truncations	CCTCTAGACTTACAAAGAAGCATGGTCAACT
CteG -200C Xba1 R	CteG truncations	CCTCTAGAACCGCCTGCTAATCCGCTG
CteG -17C FLAG SalI R	pBomb4 plasmid cloning	CCGTCGACTTACTTATCGTCGTCATCCTTGTAATC TTCTCTCATAAGATCTTTACTTT
CteG -33C FLAG SalI R	pBomb4 plasmid cloning	CCGTCGACTTACTTATCGTCGTCATCCTTGTAATC CTCTAATAGGGATAGGAAAGAAC
CteG -50C FLAG SalI R	pBomb4 plasmid cloning	CCGTCGACTTACTTATCGTCGTCATCCTTGTAATC ACGCACTTCTGCTCGAGTTCTGT
TmeA KpnI F	Ectopic expression	CCGGTACCATGAGTATTCGACCTACTAATGGGAG
TmeA NotI R	Ectopic expression	CCGCGGCCGCGTCTAAGAAAACAGAAGAAGTT
TmeA KpnI +1 F	Yeast suppressor screen	CCGGTACCAATGAGTATTCGACCTACTAATGGGA G
TmeA XbaI R	Yeast suppressor screen	CCTCTAGATTAGTCTAAGAAAACAGAAGAAGTTA TGAC
TmeA Not F	pBomb4 plasmid cloning	CCGCGGCCGCATGAGTATTCGACCTACTAATGGG AG
TmeA Flag SalI R	pBomb4 plasmid cloning	CCGTCGACTTACTTATCGTCGTCATCCTTGTAATC GTCTAAGAAAACAGAAGAAGTTATGAC
IncC NotI F	pBomb4 plasmid cloning	CCGCGGCCGCATGACGTACTCTATGTCCGATA
IncC FLAG SalI R	pBomb4 plasmid cloning	CCGTCGACTTACTTATCGTCGTCATCCTTGTAATC GCTTACATATAAAGTTTGAGGAT
CpoS SalI F	protein purification cloning	GACGCGTCGACCTTGCTAGAGCACGAAGTCGT
CpoS NotI R	protein purification cloning	GACGCGCGGCCGCTTTTTTACGACGGGATGCCTG

Table S2. Complete list of filtered AP-MS peptides for CteG.

Protein Description	Accession	Database	Avg. Score	Mass	Avg. Num. of Sig. Matches	Avg. Num. of Sig. Sequences
Actin, cytoplasmic 2	P63261	UniProt_ Human	2445	41766	77	20
Type III secretion system protein	A0A654L4P8	Chlamydia_ trachomatis _L2434Bu	1049	41172	28	15
Centrin-2	P41208	UniProt_ Human	844	19726	21	9
Putative RNA-binding protein Luc7-like 2	Q9Y383	UniProt_ Human	681	46486	20	10
Filamin-A	Q60FE5	UniProt_ Human	493	278053	20	16
Tropomodulin-3	Q9NYL9	UniProt_ Human	428	39570	13	10
Uncharacterized *C.t.* protein	A0A654L6L6	Chlamydia_ trachomatis _L2434Bu	425	68204	11	7
Peptidyl-prolyl cis-trans isomerase B	P23284	UniProt_ Human	312	23728	11	8
Girdin	A0A2R8Y7B1	UniProt_ Human	243	185366	9	7
Cleavage and polyadenylation specificity factor subunit 6	F8WJN3	UniProt_ Human	456	52238	7	4
Activated RNA polymerase II transcriptional coactivator p15	P53999	UniProt_ Human	173	14386	7	6
ATP synthase subunit alpha, mitochondrial (Fragment)	K7EK77	UniProt_ Human	131	22186	6	6
4F2 cell-surface antigen heavy chain	J3KPF3	UniProt_ Human	186	68059	5	4
Transaldolase	A0A654L6E7	Chlamydia_ trachomatis _L2434Bu	252	36138	5	4
60S acidic ribosomal protein P2	P05387	UniProt_ Human	141	11658	4	4
Molecular chaperone DnaK	A0A654L6Q8	Chlamydia_ trachomatis _L2434Bu	147	70800	4	4
FACT complex subunit SPT16	Q9Y5B9	UniProt_ Human	88	119838	4	4
Keratin, type I cytoskeletal 9	P35527	UniProt_ Human	252	62027	4	4

Signal recognition particle receptor subunit beta (Fragment)	H7C4H2	UniProt_ Human	157	17602	4	4
Peptidyl-prolyl cis-trans isomerase A	P62937	UniProt_ Human	110	18001	4	3
Importin-9	Q96P70	UniProt_ Human	91	115889	4	2
Protein disulfide-isomerase A3	P30101	UniProt_ Human	102	56747	4	4
Ribosomal protein L19	J3KTE4	UniProt_ Human	154	23233	4	3
60S ribosomal protein L7a (Fragment)	Q5T8U3	UniProt_ Human	92	21531	3	3
60 kDa chaperonin	A0A654L5L1	Chlamydia_ trachomatis _L2434Bu	62	58054	3	3
60S ribosomal protein L23a (Fragment)	H7BY10	UniProt_ Human	86	17792	3	2
60S ribosomal protein L13	P26373	UniProt_ Human	81	24247	3	3
Calmodulin-1	P0DP23	UniProt_ Human	143	16827	3	3
40S ribosomal protein S6	A2A3R5	UniProt_ Human	85	24953	3	3
Cofilin, non-muscle isoform (Fragment)	E9PP50	UniProt_ Human	102	17766	3	3
Peptidyl-prolyl cis-trans isomerase-like 4	Q8WUA2	UniProt_ Human	59	57189	3	2
Putative RNA-binding protein Luc7-like 1	Q9NQ29	UniProt_ Human	122	43701	3	2
50S ribosomal protein L16	A0A654L7G0	Chlamydia_ trachomatis _L2434Bu	134	15765	3	3
Adenosylmethionine decarboxylase	A0A5F9ZHD 5	UniProt_ Human	111	53138	3	3
Molecular chaperone GrpE	A0A654L6N2	Chlamydia_ trachomatis _L2434Bu	87	21655	3	2
Mediator of RNA polymerase II transcription subunit 1	Q15648	UniProt_ Human	181	168373	3	2
Spectrin beta chain	A0A087WUZ 3	UniProt_ Human	75	274659	3	2
Molecular chaperone DnaJ	A0A654L6M8	Chlamydia_ trachomatis _L2434Bu	93	41890	3	3
Heat shock 70 kDa protein 1B	A0A0G2JIW1	UniProt_ Human	89	70066	3	3
60S ribosomal protein L28	H0YKD8	UniProt_ Human	57	19060	3	2

Importin subunit beta-1 (Fragment)	J3QR48	UniProt_ Human	78	16388	2	2
S-adenosylmethionine synthase isoform type-2	P31153	UniProt_ Human	129	43633	2	2
HLA class I histocompatibility antigen, A alpha chain	A0A0G2JPD3	UniProt_ Human	97	44397	2	2
Dihydropteroate synthase	A0A654L9I1	Chlamydia_ trachomatis _L2434Bu	80	50230	2	2
Centrin-3	E5RJF8	UniProt_ Human	59	22409	2	2
ATP synthase subunit d, mitochondrial	F5H608	UniProt_ Human	55	8910	2	2
30S ribosomal protein S10	A0A654L6R0	Chlamydia_ trachomatis _L2434Bu	61	11862	2	2
Pre-mRNA-splicing factor 38A	Q8NAV1	UniProt_ Human	44	37453	2	2
50S ribosomal protein L21	A0A654L7I1	Chlamydia_ trachomatis _L2434Bu	62	12155	2	2
Membrane protein	A0A654LGL6	Chlamydia_ trachomatis _L2434Bu	98	17529	2	1
Catenin (Cadherin-associated protein), alpha 1, isoform CRA_a	G3XAM7	UniProt_ Human	125	92663	2	2
Albumin	A0A087WWT 3	UniProt_ Human	94	45118	2	1
Dehydrogenase	A0A654L7A9	Chlamydia_ trachomatis _L2434Bu	73	26816	2	2
Histone H1.2	P16403	UniProt_ Human	75	21352	2	2
FACT complex subunit SSRP1	Q08945	UniProt_ Human	69	81024	2	2
50S ribosomal protein L17	A0A654L6Q2	Chlamydia_ trachomatis _L2434Bu	59	16142	2	2
Spindlin interactor and repressor of chromatin-binding protein	Q9BUA3	UniProt_ Human	73	41011	2	2
60 kDa heat shock protein, mitochondrial	P10809	UniProt_ Human	39	61016	2	2
50S ribosomal protein L13	A0A654L5X5	Chlamydia_ trachomatis _L2434Bu	54	16839	2	2

Membrane-associated progesterone receptor component 1	O00264	UniProt_ Human	68	21658	2	2
30S ribosomal protein S9	A0A654L6N5	Chlamydia_ trachomatis _L2434Bu	56	14533	2	2
Vimentin	P08670	UniProt_ Human	58	53619	2	2
Calcium homeostasis endoplasmic reticulum protein	J3QK89	UniProt_ Human	61	104868	2	1
60S ribosomal protein L21	P46778	UniProt_ Human	34	18553	2	2
60S ribosomal protein L26	J3KSS0	UniProt_ Human	31	7570	2	1
Pre-mRNA 3'-end-processing factor FIP1 (Fragment)	H0Y8P7	UniProt_ Human	116	29476	2	2
Brain acid soluble protein 1	P80723	UniProt_ Human	99	22680	2	2
Serine/threonine-protein phosphatase 6 regulatory subunit 1	Q9UPN7	UniProt_ Human	107	96664	2	2
Ras-related protein Rab-5C	P51148	UniProt_ Human	89	23468	2	1
Preproteins translocase J	A0A654L770	Chlamydia_ trachomatis _L2434Bu	73	35489	2	1
60S ribosomal protein L34	P49207	UniProt_ Human	44	13284	2	1
40S ribosomal protein S12	P25398	UniProt_ Human	105	14505	2	1
60S ribosomal protein L5 (Fragment)	A0A2R8Y6J3	UniProt_ Human	42	27028	2	2
eIF-2-alpha kinase activator GCN1	Q92616	UniProt_ Human	41	292572	2	2
Uncharacterized *C.t.* protein	A0A654LIJ4	Chlamydia_ trachomatis _L2434Bu	52	32014	2	2
Importin-8	F5H815	UniProt_ Human	26	3990	2	2
